# Prevalence, Genetics, and Evolution of Porcine Astrovirus 3 in China

**DOI:** 10.1155/tbed/3170440

**Published:** 2025-07-24

**Authors:** Qingqing Wu, Jianfeng Jiang, Qingxian Li, Chaoting Xiao, Baoyu Chen, Mingkai Sun, Jiachao Xu, Shijiang Mi, Biao He, Changchun Tu, Wenjie Gong

**Affiliations:** ^1^State Key Laboratory for Diagnosis and Treatment of Severe Zoonotic Infectious Diseases, Key Laboratory for Zoonosis Research of the Ministry of Education, College of Veterinary Medicine, Jilin University, Changchun 130062, China; ^2^State Key Laboratory of Pathogen and Biosecurity, Changchun Veterinary Research Institute, Chinese Academy of Agricultural Sciences, Changchun 130122, China; ^3^College of Biology, Hunan University, Changsha 410012, China; ^4^Guangzhou Kexiang Biological Technology Co. Ltd., Guangzhou 510640, China; ^5^Jiangsu Co-Innovation Center for Prevention and Control of Important Animal Infectious Diseases and Zoonoses, Yangzhou University, Yangzhou 225009, China

**Keywords:** evolution, genotyping, PAstV3, prevalence

## Abstract

Porcine astrovirus 3 (PAstV3) is an emerging virus associated with porcine polioencephalomyelitis outbreaks and is frequently detected in the feces of both healthy and diarrheal pigs. Despite its significance, the prevalence, genetic characteristics, and evolutionary features of PAstV3 in China remain poorly understood. Here, a retrospective study of PAstV3 infection was conducted using 634 tissue or fecal specimens collected from diseased pigs across 29 regions in China between 1990 and 2024. As a result, eight tissue samples and 32 fecal samples from seven regions tested positive for PAstV3, yielding an overall positive rate of 6.3% (40/634). Through a combination of metatranscriptomics (MTT) and RT-PCR, nearly complete genome sequences of 14 PAstV3 strains and full-length open reading frame (ORF) 2 sequences of another 13 PAstV3 strains were successfully obtained. Sequence analysis revealed that these strains exhibited a sequence identity of 59.8%–100% in the capsid protein. Phylogenetic analysis demonstrated that global PAstV3 strains could be classified into four genotypes: PAstV3a–d. The intergenotype strains showed nucleotide (nt) and amino acid sequence identities of 59.7%–69.8% and 59.5%–72.0%, respectively, in the ORF2 region. Notably, PAstV3b was identified as the predominant genotype in China. Sequence comparisons further confirmed the close genetic relationship between PAstV3 and neurotropic mammalian astroviruses, suggesting the potential interspecies transmission or a shared evolutionary origin. Phylodynamic analysis estimated that the most recent common ancestor (tMRCA) of PAstV3 emerged around the year 1660 and the effective population size of PAstV3 has been continuously expanding over time.

## 1. Introduction

Astroviruses exhibit a broad host range, infecting numerous animal species across mammals and birds. Within the family *Astroviridae*, two genera have been established: *Avastrovirus* and *Mamastrovirus* (https://ictv.global/report/chapter/astroviridae/astroviridae). Members of the genus *Avastrovirus* typically cause extra-intestinal disease in infected animals [1, 2]. Conversely, the genus *Mamastrovirus* currently encompasses 19 defined species, with most causing gastroenteritis [3]. In addition, emerging evidence highlights nonenteric astrovirus infections, including encephalitis, congenital tremors, and acute respiratory disease [4]. In the 2000s, shaking mink syndrome, a neurological disorder, affected farmed minks in Denmark, Sweden, and Finland, with astrovirus detected in brain tissues [5]. Subsequently, the disease was experimentally replicated by inoculating healthy minks with brain homogenates from affected animals [5, 6]. In 2010, pyrosequencing identified astrovirus in the frontal cortex biopsy of a 15-year-old boy with X-linked gammaglobulinemia and unexplained encephalitis, suggesting the potential role of astrovirus as an encephalitis pathogen in humans [7]. Subsequently, there has been a notable increase in reported cases and epidemics of neurovirulent astroviruses in domestic mammals, including cattle, sheep, and pigs [8–12]. These findings challenge the conventional classification of astroviruses as solely enteric pathogens, indicating a broader pathogenic potential of these viruses beyond the gastrointestinal tract.

To date, five lineages of porcine astroviruses (PAstVs; PAstV1 to PAstV5) have been identified based on the diversity of their capsid protein [13]. In natural infections, PAstVs are primarily associated with diarrhea, often coexisting with other known enteric pathogens [14]. However, PAstV3 has recently garnered significant attention due to its association with encephalomyelitis. PAstV3 was first detected in the feces of a healthy pig in Canada in 2006 [15]. Subsequently, countries including the USA, Japan, Kenya, and Croatia reported PAstV3 infections in pigs, with detection in fecal samples from both healthy and diarrheal pigs at a low positive rate ranging from 0.15% to 40% [16–25]. In 2017, concurrent reports of PAstV3-associated polioencephalomyelitis outbreaks in the USA and Hungary [11, 12] highlighted its neurotropic potential. Affected pigs exhibited posterior paraplegia and occasional convulsions and PAstV3 viral loads were detected in the central nervous system (CNS) and internal organs such as lungs, tonsils, and intestines. Experimental inoculation of piglets with infectious CNS tissue homogenate recapitulated these clinical signs and pathological changes, confirming PAstV3's ability to cause neurological diseases in pigs [26]. A retrospective study of unexplained swine neurological cases in the USA from 2010 to 2017 identified 13 out of 50 cases as PAstV3-positive, with the earliest case dating back to 2010 [27]. More recently, in 2024, South Korea and Brazil reported PAstV3 detection in the CNS of weaned piglets with polioencephalomyelitis [28, 29].

In China, only a few studies have reported the detection of PAstV3 in the feces of healthy pigs in Guangxi Province, with a positive rate ranging from 0.15% to 40% [23–25]. Additionally, a high seroprevalence of 44.1% (120 out of 272 samples) was observed in Hunan and Jiangxi Provinces [30]. Considering the neurotropic pathogenicity of PAstV3, its frequent detection in fecal samples, and the scarcity of data regarding its prevalence, genetic diversity, and evolutionary characteristics, elucidating its nationwide epidemiological situation in China is of paramount importance. Therefore, we conducted a retrospective study on PAstV3 infection, utilizing archival porcine samples collected from 29 provinces over a span of 34 years and analyzed the genetics and evolution of global PAstV3 strains.

## 2. Materials and Methods

### 2.1. Clinical Samples

Between 1990 and 2024, a total of 634 samples were collected from pigs exhibiting clinical symptoms across 29 regions in China. The sample size comprised 541 internal organ samples, including lung, kidney, spleen, liver, and lymph node, and 93 samples from diarrheal pigs, consisting of feces, intestinal tissues, or swab samples. Each sample was obtained from an individual pig and stored at −80°C in our laboratory.

### 2.2. RT-PCR and Metatranscriptomics (MTT)

Samples were processed following the procedures described below. In brief, tissue samples were homogenized to prepare 10% tissue suspension in Dulbecco's Modified Eagle Medium, which were then clarified at 12,000 × *g*, 4°C for 15 min followed by filtration through 0.45 μm filters. Total RNA was extracted from the clarified supernatants using RaPure Viral RNA/DNA Kit (Magen, Guangzhou, China), followed by reverse transcription into cDNA using random primers and oligo (dT) (TaKaRa, Dalian, China). The resulting cDNA served as template for PCR detection of PAstV3 and for amplifying the open reading frame ORF2 and ORF1b genes, with primers targeting conserved regions used (Table 1). The PCR mixture contained 12.5 μL of 2× Master Mix (Tiangen, Beijing, China), 1 μL each of forward and reverse primers (10 μM), 2 μL of cDNA, and 8.5 μL of DNase-RNase free water (TaKaRa, Dalian, China). The PCR cycles conditions were set as follows: 95°C for 3 min, followed by 36 cycles of 95°C for 30 s, 56°C for 30 s, 72°C for 45 s, and a final extension at 72°C for 10 min. After confirmation by agarose gel electrophoresis, positive PCR amplicons were directly sequenced in an ABI 3730XL sequencer.

PAstV3-positive samples confirmed by RT-PCR were subjected to MTT analysis. In brief, the ribosomal RNA in the total RNA extracted using Trizol reagents was depleted using the Epicentre Ribozero rRNA Removal Kit (Epicentra Biotechnologies, Madison, USA). The remaining RNA was then precipitated by ethanol and used to construct RNA sequencing library, following the manufacturer's protocol of the NEBNext Ultra Directional RNA Library Prep Kit for Illumina (NEB, USA). Subsequently, sequencing was performed on the Illumina platform NovaSeq6000 (Novogene, Tianjin, China), generating 6 Gb of clean data with 150 bp paired-end reads for each sample. After quality control and host genome sequence removal using BWA software, virus-like reads mapping to the Protein Reference Viral Database were *de novo* assembled into contigs using both Diamond v2.0.11 and SPAdes genome assembler v3.14.1 [31, 32].

### 2.3. Amplification, Sequencing, and Genetic Analysis of PAstV3 Genome

To obtain complete genome sequences of the PAstV3 strains, PCR was used to fill the gaps between different genomic fragments. Primers were designed based on the obtained contigs. Following agarose gel electrophoresis, the PCR amplicons were sequenced using an ABI 3730XL sequencer. ORF identification and sequence identity analysis of the obtained genome sequences were conducted using the DNASTAR Lasergene software package [33]. Multiple sequence alignments of amino acid sequences of the PAstV3 capsid proteins were analyzed using the CLC Sequence Viewer v8.0 (Qiagen, Germany). Phylogenetic analysis was performed based on the capsid protein sequences of PAstV3 strains identified in this study, along with the reference mammalian astrovirus strains deposited in GenBank. Maximum-likelihood (ML) trees were constructed using MEGA 7.0, applying 1000 bootstrap replicates and the best fit substitution model [34].

### 2.4. Evolutionary Dynamic Analysis

The Bayesian Markov chain Monte Carlo (MCMC) methods implemented in the BEAST package (v1.10.4) [35] were used to estimate the tMRCA, evolutionary rates as well as the effective population size of PAstV3. For this analysis, a dataset comprising deduced amino acid sequences of PAstV3 ORF1b (encoding RdRp) was assembled, including sequences retrieved from GenBank and those generated in this study. Prior to the Bayesian analysis, PhyloSuite v1.2.3 [36] was employed to determine the best-fit model for amino acid substitution based on the lowest Bayesian information criterion (BIC) scores. The JTT + G4 model was selected, along with an uncorrelated log-normal relaxed molecular clock model and a Bayesian skyline model as the coalescent tree prior. The MCMC analysis was run for 400 million generations, with sampling occurring every 40,000 generations. The resulting log file was analyzed using Trace v1.6 (http://tree.bio.ed.ac.uk/software/tracer/) to ensure that all effective samples sizes exceeded 200. TreeAnnotator v1.10.4 was then used to construct a maximum clade credibility (MCC) tree, excluded the first 10% of the trees as burn-in. Finally, the phylogenetic tree with median node heights was visualized using FigTree v1.4.3 (http://tree.bio.ed.ac.uk/software/figtree/).

## 3. Results

### 3.1. Epidemiological Situations of PAstV3 Infection in China

**Figure 1:**
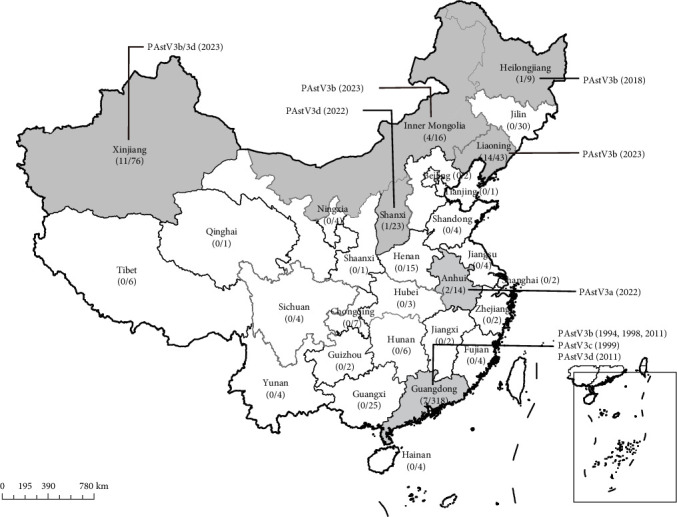
Geographical distribution of PAstV3-positive samples in China. The archival samples used for the molecular epidemiology of PAstV3 were collected from 29 regions across China between 1990 and 2024. Regions with PAstV3-positive samples are shaded in gray. Numbers within brackets denote the PAstV3 detection rates. Information indicated by the extension lines specifies the PAstV3 genotype and the collection date of the positive samples.

RT-PCR analysis revealed that PAstV3 was detected in 40 out of 634 samples (6.3%), including eight tissue samples and 32 diarrheal samples (Table 2). Notably, the detection rate of internal organs was 1.5% (8/541), which is significantly lower than the 34.4% (32/93) observed in fecal samples. These positive samples were distributed across seven regions, including Guangdong (7), Anhui (2), Heilongjiang (1), Inner Mongolia (4), Liaoning (14), Shanxi (1), and Xinjiang Provinces (11) (Figure 1). Among the eight PAstV3-positive tissue samples, the diseased pigs were concurrently infected with pathogens such as classical swine fever virus (CSFV), porcine circovirus 2 (PCV2), or porcine reproductive and respiratory virus (PRRSV). For the 32 PAstV3-positive diarrheal samples, coinfections with enteric viruses, including porcine epidemic diarrhea virus (PEDV), transmissible gastroenteritis virus (TGEV), and porcine rotavirus A (PoRVA) were identified. In addition, the earliest documented PAstV3 infection case in China dates back to 1994 in Guangdong province, which is 12-year earlier than the previously reported earliest case in Canada [15].

### 3.2. Genomic Characterization of PAstV3 Strains From China

Among the PAstV3-positive samples, 22 of them including seven tissue samples and 15 fecal or anal swab samples were subjected to MTT analysis, with 0–249,461 reads annotated to PAstV3 in each sample. In the tissue samples, reads corresponding to CSFV, PCV2, and PRRSV accounted for the largest proportion, with PAstV3 reads showing relatively low abundance. In the sequenced diarrheal samples, the most abundant reads were assigned to enteric viruses, such as PEDV, PoRVA, and enterovirus G, while PAstV3 reads represented only a minor fraction. Through MTT, 42 PAstV3 contigs were generated, ranging from 231 to 6527 nucleotide (nt) in length. Nearly complete genome sequences of nine strains were obtained. To acquire the whole-genome sequences of the remaining strains, PCR was performed using primers designed based on the contigs. Eventually, nearly complete genome sequences (6180–6527 nt) of 14 PAstV3 strains and ORF2 gene sequences of other 13 PAstV3 strains were successfully obtained (Table 3). The obtained PAstV3 strains exhibited typical astrovirus genomic structures, consisting of three partially overlapping ORFs flanked by UTRs and poly (A), arranged in the order 5' UTR-ORF1a-ORF1b-ORF2-3' UTR-poly (A). ORF1a and ORF1b are conserved genes, with all studied strains have lengths of 2535 nt and 1530 nt, respectively. (Table 3). Sequence comparison of ORF1a and ORF1b revealed that the PAstV3 strains obtained in this study shared 84.5%–94.6%/92.2%–98.3%, and 86.3%–95.2%/92.4%–100.0% nt/aa sequence identities with the reference strains, respectively. The PAstV3 ORF2 genes varied in length from 2268 to 2301 nt and shared 60.4%–93.6%/59.0%–99.3% nt/aa sequence identities with the reference PAstV3 strains, indicating that ORF2 is more variable compared to ORF1a and ORF1b.

### 3.3. Genetic and Evolutionary Characteristics of Global PAstV3 Strains

**Figure 2:**
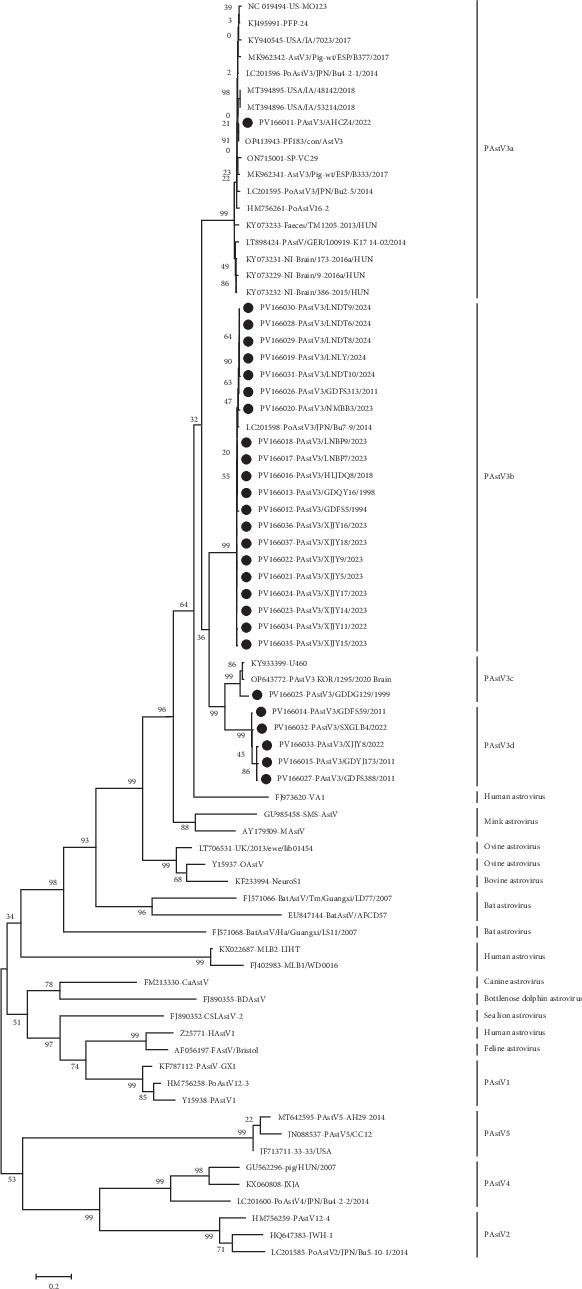
Phylogenetic tree based on the aa sequences of the capsid proteins of PAstV3 strains. A total of 27 PAstV3 strains identified in this study, along with 48 reference mammalian astroviruses, were included in the analysis. The phylogenetic tree was constructed using MEGA v7.0, employing 1000 bootstrap replicates and the best-fit substitution model (LG + G + I). PAstV3 strains identified in this study were marked with black dots (•).

A ML phylogenetic tree was constructed based on the aa sequences of the capsid proteins deduced from ORF2 of 27 obtained PAstV3 strains, 20 selected PAstV3 strains available from GenBank, and 24 prototypical strains of other mammalian astroviruses. The analysis revealed that global PAstV3 strains could be grouped into four genotypes: PAstV3a, PAstV3b, PAstV3c, and PAstV3d (Figure 2). PAstV3a, composed of the majority of strains, is widely distributed across multiple countries, which were predominantly detected in brains and fecal samples and are dominant in the United States, Japan, Canada, and several European nations. In this study, only one PAstV3a strain (PAstV3/AHCZ4/2022) was identified from the fecal sample of a diarrheal piglet in Anhui province in 2022, sharing 94.6%–99.3% aa sequence identity of the capsid protein with all PAstV3a reference strains. PAstV3b comprises strains circulating in Asian countries, primarily China and Japan. Twenty-seven PAstV3 strains identified in this study belonged to PAstV3b, highlighting its dominance in China. Two PAstV3b strains were detected in CSFV-positive samples collected from Guangdong in 1994 and 1998, while the remaining strains were identified between 2011 and 2024. Pairwise comparison showed that PAstV3b strains collected in 1990s exhibited high homology (97.5%–99.5% nt in ORF2) with other strains, suggesting that PAstV3b strains circulating in China are relatively conserved. Additionally, a single PAstV3b strain was detected in Japan from the feces of a healthy pig, showing 97.8%–98.7% aa identity of capsid protein with Chinese PAstV3b strains. Only three strains, sharing 90.5%–98.2% aa identity of capsid protein, were classified into PAstV3c. These strains were detected in a pig with encephalitis in South Korea, one healthy pig in Uganda, and a PCV2-positive kidney tissue sample collected from Guangdong in 1999. All five PAstV3d strains were identified in China in this study, detected in 2011 from Guangdong (3), and in 2022 from Xinjiang (1) and Shanxi (1), with 95.0%–99.3% aa identity of the capsid protein among them.

Further genome sequence comparison indicated that all PAstV3 strains shared 65.1%–100% nt sequence identity and the intragenotype strains exhibited close relationships (>85.0%). Among the three ORFs, ORF2 was the most variable region. Intergenotype strains showed nt and aa sequence identities of 59.7%–69.8% and 59.4%–72.0%, respectively, whereas the intragenotype strains had higher sequence identities (>81.8% nt and >90.5% aa). In contrast, ORF1a and ORF1b showed no significant differences between intergenotype and intragenotype strains, with nt/aa identities of 84.5%–91.0%/94.4%–99.4% and 70.1%–97.1%/78.5%–99.6% for intergenotype strains and 84.2%–100%/92.1%–100% and 70.9%–100%/79.6%–100% for intragenotype strains, respectively. Therefore, the ORF2-encoded capsid proteins of PAstV3 strains can serve as an effective target for genotyping, with a 28.0% aa-level genetic difference threshold distinguishing different genotypes.

**Figure 3:**
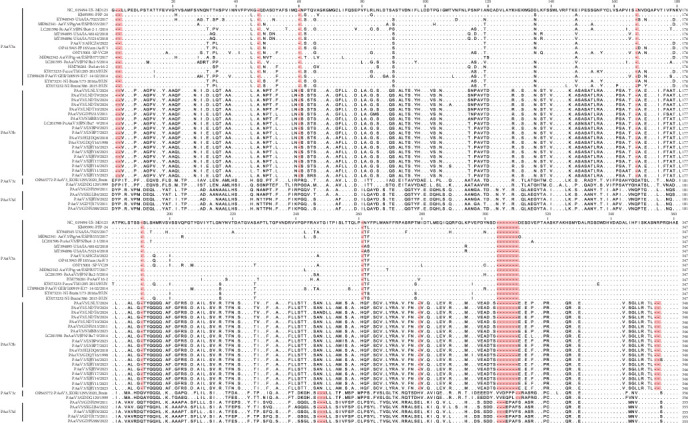
Multiple sequence alignment of the C-terminal region of PAstV3 capsid protein. A sequence comparison of the C-terminal region (409–755 aa) of the capsid protein from 45 PAstV3 strains was performed, using the strain US-MO123 (GenBank accession number: NC019494) as the reference.

The C-terminal domain of the capsid protein, a hypervariable region, forms the virion spike and determines protein antigenic diversity. As depicted in Figure 3, different genotype strains displayed distinct characteristics in this region (at 409–755 aa relative to the prototype strain US-MO123). Compared with PAstV3a strains, PAstV3b strains had two insertions (A/T at position 452 and H at 662) and two deletions (H/E/M at 680–681 and NR at 748–749). PAstV3c and PAstV3d shared similar patterns, featuring five insertions (F(Y)P at 409, T/S/N at 452, Q/H at 570, Q at 593, and H/P/L at 662) and one deletion (VTG at 649–651). Additionally, PAstV3c and PAstV3d carried one insertion (VVDPP/VVEQP at 704) and two insertions (P at 464 and EPAF/EPVF at 704), respectively. Despite the diversity, five conserved residue regions were identified across four genotypes: ^447^V (I)FPV^450^, ^545^WLV (A)RF (L)^549^, ^715^SKF (L)AK^719^, ^725^ALRDSD (E)WDHVDAD^737^, and ^751^RGHAE^755^.

As depicted in Figure 2, the PAstV3 clade clustered with other mammalian astroviruses from mink, human, sheep, and bats, but diverged from the clades formed by the other four PAstV species (PAstV1, PAstV2, PAstV4, and PAstV5). This phylogenetic pattern suggests that PAstV3 may share a common ancestor with these mammalian astroviruses and that cross-species transmission events likely occurred among them. Sequence identity comparisons of different genomic regions further supported the closer phylogenetic relationship between PAstV3 and other mammalian astroviruses (Table 4). For the variable ORF2 gene, PAstV3 strains shared 35.9%–42.6% nt and 19.2%–26.2% aa identity with the other four PAstVs, significantly lower than the identities with mammalian astroviruses (38.5%–55.4% nt and 20.9%–50.6% aa). For the relative conserved ORF1a and ORF1b, the nt/aa identities between PAstV3 strains and the other four PAstVs were 39.7%–46.9%/23.2%–29.9% and 52.0%–59.8%/44.0%–54.2%, respectively, also lower than those with mammalian astroviruses (45.0%–57.3%/25.2%–48.3% nt and 55.3%–64.2%/50.4%–65.9% aa).

### 3.4. Evolutionary Dynamics of Global PAstV3 Strains

**Figure 4:**
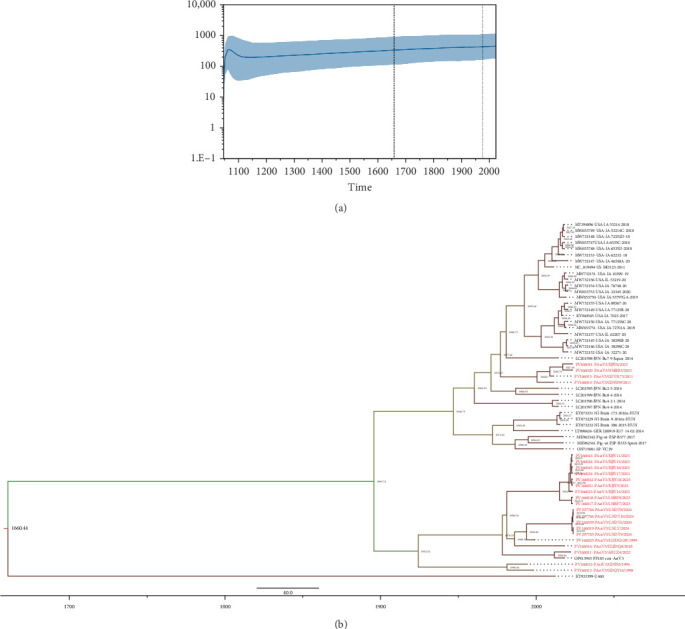
Bayesian skyline plot and maximum clade credibility (MCC) tree based on 59 amino acid sequences of ORF1b (479 aa). (A) Bayesian skyline plot showing changes in the effective population size of PAstV3 strains over time. The *γ*-axis of the skyline plot represents the effective population size. The thick blue line indicates the median estimate and the shaded area represents the 95% highest posterior density (HPD) interval. (B) The MCC tree was constructed using the JTT + G4 substitution model and an uncorrected log-normal relaxed clock. The MCMC analysis was run with a total chain length of 4 × 10^9^ generations and sampled every 4 × 10^4^ times. Strains obtained in this study are marked in red.

In this study, amino acid sequences of ORF1b encoding the RNA-dependent RNA polymerase were used for analyzing the phylodynamic characteristics of PAstV3 strains [37–40]. Phylodynamic analysis of the effective population size over time indicated that PAstV3 underwent a rapid expansion following its initial appearance in 1047.19, reaching a peaked around 1076.60. Subsequently, the population size steadily increased from 1135.81 onwards (Figure 4a). Bayesian MCMC analysis estimated that the tMRCA of PAstV3 emerged in 1660.44, with a 95% highest posterior density (HPD) interval of 1047.19–1978.06. As illustrated in Figure 4b, the MCC tree revealed three clusters, in which the strain from Uganda (KY933399, U460) was identified as the earliest among the analyzed strains, potentially representing an ancestral lineage of PAstV3. The remaining strains diverged into two major branches: the upper branch, established in 1946.79, consisted of strains from Japan, the United States, Hungary, and four strains characterized in this study. The other branch comprised exclusively Chinese strains, with a common ancestor dating back to 1924.24. The mean evolutionary rate of PAstV3 was estimated at 5.35 × 10^−4^ (95% HPD: 1.18 × 10^−4^−1.10 × 10^−3^) substitution/site/year.

## 4. Discussion

PAstV3 has recently garnered attention due to its potential neuroinvasive properties and frequent detection in piglet diarrhea. Understanding the prevalence and genetic diversity of PAstV3 in major pig-producing regions across China is crucial for the sustainable development of the pig industry. In the present study, a retrospective investigation was conducted with the archival diseased samples collected across 29 regions in China over 34 years, with a low prevalence rate (6.3%, 40/634) detected. With the large sample size and wide geographical distribution, this finding represents the natural infections of this virus in China, and the detection rate aligns with previous reports, which have documented PAstV3 detection in several European, African, North American, and Asian countries, with prevalence rates ranging from 0.15% to 40% [16–25, 27]. Notably, the positive rate of PAstV3 in fecal samples from diarrheic pigs (34.4%, 32/93) was significantly higher than that in tissue samples (1.5%, 8/541). High detection rates of up to 40% in diarrheic pigs have also been reported previously in China [25]. The elevated prevalence in the feces of diarrheic pigs suggests the possible contribution of PAstV3 infection to the onset and progression of piglet diarrhea. Additionally, PAstV3 was detected in several tissue samples, including lymph nodes, liver, and kidney (Table 2), further confirming the virus broad tissue tropism. Unfortunately, no brain samples were collected from PAstV3-positive pigs displaying diarrhea and other clinical signs, precluding our ability to determine the neurotropism of these PAstV3 strains, thus, investigations into PAstV3-associated polioencephalomyelitis should be prioritized in the future with diseased pigs exhibiting either nervous disorder or diarrhea. Furthermore, coinfection of PAstV3 with other viral pathogens was detected in all PAstV3-positive samples identified in this study (Table 2), including CSFV, PCV2, PRRSV, PEDV, PoRVA, and enterovirus G, which was consistent with the results of previous report that coronaviruses and rotaviruses were frequently observed to be coinfected with PAstV3 in the fecal samples [14]. Nevertheless, based on HTS data (published elsewhere), the abundance of PAstV3 in both tissue samples and fecal samples was much lower than that of other viruses, further pending the determination of the pathogenicity of PAstV3 strains in the disease development of piglet diarrhea. To comprehensively elucidate the pathogenesis and potential zoonotic risks of PAstV3, *in vitro* virus isolation should be performed, which is essential for evaluating viral virulence and other etiological properties.

Among the proteins encoded by the three ORFs of PAstV3, the capsid protein is the most variable, which consists of a conserved N-terminus and a hypervariable C-terminus. This structural protein is predicted to mediate the interaction between the virus and host cells and is commonly used for astrovirus genotyping [41, 42]. Based on the genetic diversity of the capsid protein, global PAstV3 strains were initially classified into four genotypes (PAstV3a–d) with intergenotypic genetic distance exceeded 28.0% and each genotype displays distinct insertion and deletion patterns in the capsid protein, as well as unique geographical distribution characteristics. Notably, PAstV3a is widely distributed across numerous European and American countries, as well as Japan, and only one PAstV3a strain was detected in China. This genotype includes both neurotropic strains and those isolated from diarrheic pigs. Thus, further research is required to evaluate the pathogenicity of PAstV3a strains in piglet diarrhea. In contrast, in China, PAstV3b is the predominant genotype, the strains of this genotype showed varying spatial and temporal distribution patterns across the country, and one Japanese strain has also been identified. These findings suggest that PAstV3a and PAstV3b have different evolutionary origins and the international transmission of PAstV3 may be attributed to the import of breeding pigs. As illustrated in Figure 2, PAstV3 strains are closely related to human/mink neurotropic astroviruses, but distantly related to the other four PAstVs, suggesting that PAstV3 shares a common ancestor with mammalian neurotropic astroviruses rather than other PAstVs, which aligns with previous research findings [20]. Moreover, evolutionary analysis based on the conserved RdRp sequences revealed that the effective population size of ancient PAstV3 has been continuously expanding. Therefore, surveillance efforts for this pathogen need to be strengthened to confirm the involvement of PAstV3 in the development of viral diarrhea, polioencephalomyelitis, and other diseases.

## 5. Conclusions

This study revealed the epidemiological situation of PAstV3 infections in China, with the earliest case dating back to 1994. Global PAstV3 strains exhibit substantial genetic diversity and can be categorized into four genotypes, each demonstrating distinct geographical distribution patterns. Given the high heterogeneity and potential pathogenicity of PAstV3, continuous surveillance of PAstV3 infections is essential, especially through sampling from the nervous system and intestines. Additionally, greater emphasis should be placed on elucidating their clinical significance and viral pathogenic mechanisms in order to effectively prevent and control PAstV3-associated diseases.

## Figures and Tables

**Table 1:** Primers of PAstV3 used for detection or amplification of ORF1b and ORF2.

Primer	Sequence (5′-3′)	Genome position^a^	Amplicon length (bp)
PAstV3/test/WF	YGGTTGCACGCACYAAGYT	104–122	643/804
PAstV3/test/NF	CCTTACTTCTATGACTTTGG	145–164	
PAstV3/test/WR	ACRACTGATGGCATATCCAT	889–908	
PAstV3/test/NR	AARAAGCCAACCATTGTTGC	769–788	

PAstV3/ORF1b/F	GCCCATTACCTACTCC	2546–2561	1551/1598
PAstV3/ORF1b/NR	GGCTTRTCACCAGCCAT	4081–4097	
PAstV3/ORF1b/WR	CCTTGGCAACCTCCTT	4129–4144	

PAstV3/ORF2/WF	AGCGGAGACTAACAAGCTT	3824–3842	2382/2537
PAstV3/ORF2/NF	CAAGATGTGATGACCCTCT	3876–3894	
PAstV3/ORF2/WR	TGTACCCTCGTTCCTACTC	6343–6361	
PAstV3/ORF2/NR	GAGAGCATCATACATGCTG	6240–6258	

^a^US-MO123 (GenBank accession number: NC 019494).

**Table 2:** The information of the 40 PAstV3-positive samples.

Sample name	Ages (days)	Time	Location	Tissue type	Clinical diagnosis	Clinical symptoms
GDFS5	NA	1994	Guangdong	Lymph node	CSFV^+^	NA
GDQY16	NA	1998	Guangdong	Liver	CSFV^+^	NA
GDYJ173	NA	2011	Guangdong	Kidney	CSFV^+^	NA
HLJDQ8	NA	2018	Heilongjiang	Liver	CSFV^+^	NA
GDDG129	NA	1999	Guangdong	Kidney	PCV2^+^	NA
GDFS59	NA	2011	Guangdong	Kidney	PCV2^+^	NA
GDFS313	NA	2011	Guangdong	Kidney	PRRSV^+^	NA
GDFS388	NA	2011	Guangdong	Kidney	PRRSV^+^/PCV2^+^	NA
NMBB1	7–23	2023	Inner Mongolia	Feces	PEDV^+^	Diarrhea
NMBB2	7–23	2023	Inner Mongolia	Feces	PEDV^+^	Diarrhea
NMBB3	7–23	2023	Inner Mongolia	Feces	PEDV^+^/TGEV^+^	Diarrhea
NMBB4	7–23	2023	Inner Mongolia	Feces	ND	Diarrhea
XJJY5	19	2023	Xinjiang	Feces	PEDV^+^	Diarrhea
XJJY8	60	2023	Xinjiang	Feces	PEDV^+^	Diarrhea
XJJY9	13	2023	Xinjiang	Feces	PEDV^+^	Diarrhea
XJJY10	20	2023	Xinjiang	Feces	PEDV^+^	Diarrhea
XJJY11	13	2023	Xinjiang	Feces	PEDV^+^	Diarrhea
XJJY13	22	2023	Xinjiang	Feces	PEDV^+^	Diarrhea
XJJY14	26	2023	Xinjiang	Feces	PEDV^+^	Diarrhea
XJJY15	14	2023	Xinjiang	Feces	PEDV^+^	Diarrhea
XJJY16	22	2023	Xinjiang	Feces	PEDV^+^	Diarrhea
XJJY17	22	2023	Xinjiang	Feces	PEDV^+^	Diarrhea
XJJY18	22	2023	Xinjiang	Feces	PEDV^+^	Diarrhea
AHCZ4	18	2022	Anhui	Feces	ND	Diarrhea
AHCZ10	22	2022	Anhui	Feces	PEDV^+^/PoRVA^+^	Diarrhea
LNBP1	3	2023	Liaoning	Feces	ND	Diarrhea
LNBP2	3	2023	Liaoning	Feces	PoRVA^+^	Diarrhea
LNBP7	3	2023	Liaoning	Feces	PoRVA^+^	Diarrhea
LNBP8	3	2023	Liaoning	Feces	ND	Diarrhea
LNBP9	3	2023	Liaoning	Feces	PoRVA^+^	Diarrhea
SXGLB4	NA	2022	Shanxi	Feces	PoRVA^+^	Diarrhea
LNDT1	5–7	2024	Liaoning	Anal swab	PoRVA^+^	Diarrhea
LNDT2	5–7	2024	Liaoning	Anal swab	PoRVA^+^	Diarrhea
LNDT3	5–7	2024	Liaoning	Anal swab	PoRVA^+^	Diarrhea
LNDT4	5–7	2024	Liaoning	Anal swab	PoRVA^+^	Diarrhea
LNDT6	5–7	2024	Liaoning	Anal swab	PoRVA^+^	Diarrhea
LNDT7	5–7	2024	Liaoning	Anal swab	PoRVA^+^	Diarrhea
LNDT8	5–7	2024	Liaoning	Anal swab	PoRVA^+^	Diarrhea
LNDT9	5–7	2024	Liaoning	Anal swab	PoRVA^+^	Diarrhea
LNDT10	5–7	2024	Liaoning	Anal swab	PoRVA^+^	Diarrhea

*Note:* NA, the information is not available. ND, the detection results are negative for common diarrhea viruses including PEDV, TGEV, PDCoV, and PoRVA.

**Table 3:** The genomic information of 14 Chinese PAstV3 strains identified in this study.

Strains	Tissue	Genome (nt)	ORF1a nt/aa length (location)	ORF1b nt/aa length (location)	ORF2 nt/aa length (location)
PAstV3/AHCZ4/2022	Feces	6416	2535/844 (19–2553)	1530/509 (2550–4079)	2268/755 (4069–6336)
PAstV3/GDFS5/1994	Lymph node	6180	2518/838^a^ (1–2518)	1530/509 (2515–4044)	2147/715^b^ (4034–6180)
PAstV3/GDQY16/1998	Liver	6435	2535/844 (17–2551)	1530/509 (2548–4077)	2277/758 (4067–6343)
PAstV3/GDFS59/2011	Kidney	6375	2535/844 (28–2562)	1530/509 (2559–4088)	2298/765 (4078–6375)
PAstV3/GDYJ173/2011	Kidney	6374	2535/844 (27–2561)	1530/509 (2558–4087)	2298/765 (4077–6374)
PAstV3/HLJDQ8/2018	Liver	6415	2535/844 (16–2550)	1530/509 (2547–4076)	2277/758 (4066–6342)
PAstV3/LNBP7/2023	Feces	6419	2535/844 (25–2559)	1530/509 (2556–4085)	2277/758 (4075–6351)
PAstV3/LNBP9/2023	Feces	6413	2535/844 (16–2550)	1530/509 (2547–4076)	2277/758 (4066–6342)
PAstV3/NMBB3/2023	Feces	6434	2535/844 (15–2549)	1530/509 (2546–4075)	2277/758 (4065–6341)
PAstV3/XJJY5/2023	Feces	6411	2535/844 (16–2550)	1530/509 (2547–4076)	2277/758 (4066–6342)
PAstV3/XJJY9/2023	Feces	6415	2535/844 (17–2551)	1530/509 (2548–4077)	2277/758 (4067–6343)
PAstV3/XJJY14/2023	Feces	6413	2535/844 (17–2551)	1530/509 (2548–4077)	2277/758 (4067–6343)
PAstV3/XJJY17/2023	Feces	6527	2535/844 (11–2545)	1530/509 (2645–4174)	2277/758 (4164–6440)
PAstV3/LNLY/2024	Feces	6407	2535/844 (11–2545)	1530/509 (2542–4071)	2277/758 (4061–6337)

^a, b^Partial ORF sequence encoding the N-terminus of pp1a and the C-terminus of capsid protein of PAstV3/GDFS5/1994 is not available.

**Table 4:** Percentage of sequence identity between PAstV3 strains and other mammal astroviruses in different genomic regions.

Species								
Region	Human astrovirus	Mink astrovirus	Bovine astrovirus	Ovine astrovirus	PAstV1	PAstV2	PAstV4	PAstV5
Genome	45.9–57.3	54.4–56.4	43.7–54.4	52.4–54.8	46.7–48.0	44.0–46.4	40.9–45.0	43.4–45.8
ORF1a-nt ORF1a-aa	45.0–57.3	52.9–54.5	51.4–54.2	50.9–54.2	44.3–46.8	41.4–45.7	39.7–44.5	40.3–46.9
	25.2–48.3	45.4–47.0	41.9–43.8	41.9–43.1	25.7–27.8	26.6–29.9	23.2–27.2	25.0–27.3
ORF1b-nt ORF1b-aa	55.3–64.2	60.5–63.1	59.5–62.8	59.1–61.6	55.5–57.8	55.2–59.8	54.3–57.6	52.0–55.4
	50.4–65.9	59.4–62.9	58.9–63.4	59.2–61.9	50.0–52.3	49.1–53.3	50.9–54.2	44.0–47.5
ORF2-nt ORF2-aa	38.5–55.1	49.9–55.4	38.8–52.6	47.2–51.4	38.0–42.6	36.5–42.4	35.9–42.0	37.8–42.6
	23.5–49.5	46.1–50.6	20.9–44.9	40.9–44.6	22.7–26.2	19.2–24.7	20.2–25.9	19.3–23.0

## Data Availability

All sequences of PAstV3 strains obtained in this study have been deposited in GenBank under accession numbers PV166011-PV166045 and PV297764-PV297766.
